# Long-term benzodiazepine use and risk of labor market marginalization in Finland: A cohort study with 5-year follow-up

**DOI:** 10.1192/j.eurpsy.2024.1745

**Published:** 2024-04-04

**Authors:** Heidi Taipale, Antti Tanskanen, Terhi Kurko, Tero Taiminen, Hanna Särkilä, Jari Tiihonen, Reijo Sund, Solja Niemelä, Leena Saastamoinen, Jarmo Hietala

**Affiliations:** 1 Department of Forensic Psychiatry, University of Eastern Finland, Niuvanniemi Hospital, Kuopio, Finland; 2 Department of Clinical Neuroscience, Karolinska Institutet, Stockholm, Sweden; 3 Center for Psychiatry Research, Stockholm City Council, Stockholm, Sweden; 4 School of Pharmacy, University of Eastern Finland, Kuopio, Finland; 5 Research Unit, The Social Insurance Institution, Helsinki, Finland; 6 Department of Psychiatry, University of Turku, Turku, Finland; 7 City of Turku Welfare Division, Turku City Hospital, Turku, Finland; 8 School of Medicine, University of Eastern Finland, Kuopio, Finland; 9 Department of Psychiatry, Turku University Hospital, The Wellbeing Services, County of Southwest, Finland; 10 Development and Information Services, Finnish Medicines Agency Fimea, Helsinki, Finland

**Keywords:** benzodiazepines, disability pension, labor market marginalization, long-term use, sickness absence, unemployment

## Abstract

**Background:**

Benzodiazepines and related drugs (BZDRs) are widely used in the treatment of anxiety and sleep disorders, but cognitive adverse effects have been reported in long-term use, and these may increase the risk of labor market marginalization (LMM). The aim of this study was to investigate whether the risk of LMM is associated with new long-term BZDR use compared to short-term use.

**Methods:**

This register-based nationwide cohort study from Finland included 37,703 incident BZDR users aged 18–60 years who initiated BZDR use in 2006. During the first year of use, BZDR users were categorized as long-term users (≥180 days) versus short-term users based on PRE2DUP method. The main outcome was LMM, defined as receipt of disability pension, long-term sickness absence (>90 days), or long-term unemployment (>180 days). The risk of outcomes was analyzed with Cox regression models, adjusted with sociodemographic background, somatic and psychiatric morbidity, other types of medication and previous sickness absence.

**Results:**

During 5 years of follow-up, long-term use (34.4%, N = 12,962) was associated with 27% (adjusted Hazard Ratio, aHR 1.27, 95% CI 1.23–1.31) increased risk of LMM compared with short-term use. Long-term use was associated with 42% (aHR 1.42, 95% CI 1.34–1.50) increased risk of disability pension and 26% increased risk of both long-term unemployment and long-term sickness absence.

**Conclusions:**

These results indicate that long-term use of BZDRs is associated with increased risk of dropping out from labor market. This may be partly explained by cognitive adverse effects of prolonged BZDR use, which should be taken into account when prescribing BZDRs.

## Introduction

Benzodiazepines are used in the treatment of a variety of psychiatric disorders and symptoms [1]. These include anxiety disorders, affective disorders, alcohol withdrawal, delirium, and agitated and/or aggressive behaviors associated with psychosis. Benzodiazepines and benzodiazepine-related drugs (also known as Z-drugs) are both used in the treatment of insomnia. Although benzodiazepines and related drugs (BZDRs) are effective for short-term treatment of insomnia, their use is associated with the risk of several adverse outcomes, for example, increased risk for tolerance, withdrawal symptoms, and rebound effects as well as development of dependence syndrome [1]. For these reasons, most clinical guidelines recommend BZDRs only for short-term use. In Finland, official recommendations state that BZDR treatment should be as short-term as possible and, as a rule, BZDR use should last no more than 4–12 weeks, including the gradual tapering of BZDRs [2, 3].

However, long-term use of BZDRs is common [4–7], and the debate on pros and cons of long-term use of BZDRs has been ongoing for decades. According to a review [4], the annual prevalence of benzodiazepine use in the general population varies between 5 and 24% and of users, 6–15% can be defined as long-term users, that is, using for 6 months or longer. We recently showed that of incident BZDR users, 39% became long-term users during the follow-up of up to 10 years [7]. Therefore, long-term use of BZDR remains a relevant clinical problem [8, 9].

Recently, more attention has been drawn to cognitive problems associated with long-term BZDR use in clinical patient populations [7, 10]. Although the effect of BZDR medication is often difficult or even impossible to separate from the symptoms of the underlying disorders in clinical practice, the association between long-term BZDR use and cognitive deficits is evident and also supported by recent experimental studies [11]. Cognitive problems in long-term BZDR users are seen across multiple domains of cognition [10]. Most affected domains, such as processing speed, nonverbal memory, attention, and problem-solving, are relevant for functioning and may readily affect social and working ability. Such deficits may result in decreased job performance and thus, lead to a worsened position in the labor market with significant economical, societal, and human losses both at the individual and society levels [12].

Even though BZDRs are among the most commonly prescribed psychotropics, little is known about the associations between BZDR use and labor market marginalization (LMM). A Norwegian study found that the majority of BZDR users had a stable pattern of BZDR use before and after granting a disability pension (DP), although a minority increased their use during DP [13]. In turn, in a Finnish study of retired employees, an increase in BZDR use was reported before the beginning of disability pension and a decreasing trend after retirement [14]. Tvete et al. assessed the risk of disability pension associated with BZDR use, showing lower risk for Z-drugs than BZDs [15]. There is a lack of studies assessing labor market outcomes more broadly than associations with one specific domain, such as disability pension.

In this study, we widen the scope and investigate whether long-term benzodiazepine and related drug use is associated with the risk of LMM compared to short-term use. LMM is defined as long-term unemployment, long-term sick leave, or disability pension, as in previous studies [16]. The rationale for including all of these aspects is due to the fact that pathways to LMM are multifaceted and include both the process of health selection and social causation and their interactions with each other [17–19]. We hypothesized that long-term BZDR use increases the probability of LMM among working-age persons who newly initiated BZDR use and became long-term users during their first year of use. The longitudinal design included a selected follow-up time period (2004–2010) when the labor market in Finland was relatively stable, with overall population-level employment rates around 70% among those aged 18–60 years.

## Methods

### Utilized registers

The study population was identified from the Dispensations reimbursable under the National Health Insurance Scheme register, which includes dispensing of reimbursed drugs for outpatients. All residents who had reimbursed prescription drug purchases for benzodiazepines or Z-drugs, defined as Anatomic Therapeutic Chemical (ATC) classification codes N03AE01 (clonazepam), N05BA, N05CD, N05CF, or N06CA01 (chlordiazepoxide-amitriptyline combination), during 2006 but did not have purchases during 2004–2005 were included in the base cohort, that is, new users [7]. Working-age persons (18–60 years) were included in this study (N = 86,086). Persons on any form of pension on January 1, 2006 (N = 15,691), those who died during the first year (N = 341) and those who purchased only one package of BZDRs during 2006 (N = 32,351) were excluded (as there is no certainty that these people used BZDRs at all and were not re-dispensed). After these exclusions, the final cohort consisted of 37,703 persons.

The Care Register for Health Care (data on dates of hospital admissions and discharges), Special Reimbursement Register (data on persons granted with a special refund for drugs due to specific chronic diseases), registers concerning sickness absence (>14 days), social benefits (based on basic social assistance, labor market subsidy, basic unemployment allowance, national pension, and study grants), and a register of persons who received child care benefits (maternity allowance, paternity allowance, parental allowance, and child home care allowance) were utilized. Data on disability pensions were extracted from the Finnish Centre for Pensions and Social Insurance Institution and combined with data from Earnings- and Accrual register from the Finnish Centre of Pensions, including periods of unemployment. Educational level (at the end of 2005) was derived from Statistics Finland.

### Permissions and ethical approval

The registers are maintained by the Finnish Institute for Health and Welfare (THL/1438/5.05.00/2014), the Social Insurance Institution of Finland (30/522/2013), the Finnish Centre for Pensions (ETK/SUTI21001), and Statistics Finland (TK-53-765-20) and these institutions granted permission for this study. According to Finnish legislation, no ethics committee approval or patient consent is required for de-identified register-based research.

### Exposure

Duration of continuous BZDR use was defined using the PRE2DUP method [20]. In this method, the use of each drug was modeled separately with sliding averages of daily dose and by considering stockpiling of drugs, personal purchasing regularity, and possible hospitalizations. Overlapping periods of specific BZDR drugs were then combined to derive duration of “any BZDR,” and also use of “any BZD” (N03AE01, N05BA, N05CD, N06CA01) versus “any Z-drug” (N05CF) use. Further categorization was made for BZDR type, defined as use of “anxiolytics only” (N05BA), “hypnotics only” (N05CD, N05CF) or both, and according to the longest half-life used as long-acting (diazepam, chlordiazepoxide, clonazepam, nitrazepam), medium-acting (alprazolam, lorazepam, oxazepam, temazepam, zopiclone) versus short-acting (midazolam, triazolam, zolpidem).

For this study, short-term versus long-term use were defined during the first year following the date of first purchase based on cumulative duration of use (in days). Those who used less than 180 days of the first year were categorized as short-term users, and those who used 180 days or longer were defined as long-term users. The cut-off of 6 months was chosen based on a previous systematic review [4]. The status was not updated during the follow-up. This categorization was used as an exposure variable in the models. In sensitivity analyses, we censored follow-up to change in exposure status, that is change from long-term use to short-term use or vice versa, or to non-use of BZDRs.

### Measurement of the main outcome

The main outcome was LMM. LMM was defined as receipt of disability pension, allowance for long-term sickness absence (>90 days), or long-term unemployment (>180 days), whichever occurred first. Data for outcome events was combined from registers of sickness absence (Social Insurance Institution), disability pensions (Social Insurance Institution, Finnish Centre of Pensions), and Earnings and Accrual register (Finnish Centre of Pensions, unemployment). Each of these events included in the LMM was also analyzed separately. For disability pensions, also the main diagnosis was analyzed according to ICD-10 first character from A to Z and separately for subcategories of Mental, Behavioral, and Neurodevelopmental disorders (category F).

### Covariates

Comorbidities measured at the time of BZDR initiation were derived from Special Reimbursement (special reimbursements granted since 1972 and before BZDR initiation) and Care Register for Health Care (diagnosis during 2 years before initiation). These included cardiovascular diseases (hypertension, coronary artery disease, chronic heart failure, stroke), diabetes, rheumatoid arthritis, asthma/COPD, cancer, inflammatory bowel disease, epilepsy, schizophrenia, bipolar disorder, depression, anxiety disorder and substance use disorder. Use of other medications for mental health problems and pain conditions was measured 30 days before BZDR initiation as use of antidepressants (ATC N06A), opioids (N02A), non-opioid analgesics (M01A, N02BE01), and muscle relaxants (M03B). We defined receipt of social benefits and sick leaves during the previous year before initiation from the registers of Social Insurance Institution and educational level from Statistics Finland. For the description of the study population, persons were categorized as being employed during the previous year before BZDR initiation if they received income from work based on the Accrual register.

### Statistical analyses

The follow-up for outcomes started for each individual 1 year after the first purchase of BZDRs, that is, after the definition period for short- versus long-term use ended. The follow-up ended to first outcome event, after 5 years, death, 65 years of age (after which person is not at risk of LMM anymore), or granted disability pension when it was not included in the analyzed outcome measure (as the person is not at risk for other outcomes either after that).

Risk of outcomes was analyzed with Cox regression models. Both unadjusted and adjusted analyses were conducted and proportional hazard assumption was assessed by plotting survival curves. Cumulative incidence of outcomes, by taking into account on competing risks, namely mortality, was estimated with R (ver 4.1.1) cmprisk (ver 2.2.11) package cuminc function, and other statistical analyses were performed with SAS statistical software, version 9.4 (SAS Institute).

As sensitivity analysis, the population were stratified according to sex, age at initiation, employment at baseline (yes vs. no), and by initial BZDR (started with benzodiazepines only vs. Z-drugs only vs. with both). Lastly, sensitivity analyses by additionally censoring to exposure group change (short-term vs. long-term vs. non-use) were conducted.

## Results

The study identified 37,703 working-age persons who initiated BZDR use in 2006 and had at least two purchases during the first year after initiation. Based on use patterns during the first year, 34.4% were classified as long-term users and the rest as short-term users (Table 1). Long-term users were more likely to be men, had low educational levels and were less likely to be employed at the initiation. They were also more likely to be prescribed only benzodiazepines, and they received their first BZDR prescription more often from psychiatry. Long-term users had higher prevalence of any psychiatric comorbidity than short-term users, whereas somatic conditions were somewhat equally distributed.

**Table 1.** Distribution of sociodemographic factors, comorbidities and other drug use for short-term versus long-term users

	Short-term users N = 24,741 % (N)	Long-term users N = 12,962 % (N)
Men	40.6 (10,045)	49.4 (6,403)
Mean age, years (SD)	42.5 (11.0)	42.5 (11.3)
Age categories, years		
18–29	17.1 (4,239)	18.3 (2,367)
30–39	22.0 (5,437)	20.5 (2,655)
40–49	30.4 (7,516)	29.5 (3,823)
50–60	30.5 (7,549)	31.8 (4,117)
Education		
High (university–level)	19.6 (4,836)	13.3 (1,729)
Medium	58.6 (14,491)	55.5 (7,196)
Low (≤9 years)	21.9 (5,414)	31.1 (4,037)
Labor market factors		
Receipt of social benefits	21.6 (5,349)	33.9 (4,390)
Previous sickness absence	24.9 (6,165)	26.1 (3,378)
Employed at start of follow–up	76.3 (18,881)	61.0 (7,907)
Comorbidities		
Any somatic comorbidity^a^	18.0 (4,462)	19.4 (2,510)
Cardiovascular disease	8.2 (2,038)	9.6 (1,248)
Diabetes	2.0 (493)	2.5 (324)
Rheumatoid arthritis	1.6 (390)	1.5 (198)
Asthma/COPD	5.6 (1,373)	5.5 (718)
Cancer	2.1 (507)	2.4 (308)
Inflammatory bowel disease	1.0 (243)	0.9 (111)
Any psychiatric comorbidity^b^	11.6 (2,861)	17.6 (2,286)
Schizophrenia	1.5 (378)	2.5 (320)
Bipolar disorder	1.0 (246)	1.4 (184)
Depression	4.4 (1,092)	6.0 (778)
Anxiety disorder	2.9 (727)	4.3 (559)
Substance use disorder	4.6 (1,140)	8.6 (1,116)
Specialty of the initial prescriber		
Neurology	3.2 (794)	3.4 (438)
Psychiatry	9.4 (2,314)	14.2 (1,839)
Occupational medicine	15.1 (3,730)	10.3 (1,338)
General medicine/no specialty	53.3 (13,192)	55.7 (7,225)
Other specialty	19.0 (4,711)	16.4 (2,122)
BZDR used at initiation		
Benzodiazepines only	32.2 (7,966)	48.9 (6,337)
Z–drugs only	54.0 (13,362)	31.5 (4,087)
Both benzodiazepines and Z–drugs	13.8 (3,413)	19.6 (2,538)
BZDR type used at initiation^c^		
Anxiolytic only	25.4 (6,278)	36.5 (4,732)
Hypnotic only	58.8 (14,540)	38.4 (4,978)
Both anxiolytic and hypnotic	13.7 (3,394)	21.1 (2,729)
Half-life of BZDR		
Short-acting	22.9 (5,673)	9.4 (1,215)
Medium-acting	57.5 (14,229)	59.0 (7,653)
Long-acting	19.6 (4,839)	31.6 (4,094)
Duration of BZDR use during the initial year, days		
Mean (SD)	85.4 (35.9)	313.0 (65.6)
Median (IQR)	82 (53–101)	365 (253–365)
Other medication use		
Antidepressant use	22.5 (5,575)	28.7 (3,716)
Opioid analgesic use	1.5 (363)	2.2 (280)
Other analgesic use	16.9 (4,191)	16.3 (2,106)
Muscle relaxant use	4.3 (1,057)	3.9 (509)

a Any somatic comorbidity refers to the ones listed in the table, namely cardiovascular disease, diabetes, asthma/COPD, rheumatoid arthritis, cancer, and inflammatory bowel disease.

b Any psychiatric comorbidity refers to the ones listed in the table, namely schizophrenia, bipolar disorder, depression, anxiety disorder, and substance use disorder.

c Clonazepem not categorized to either group.

Supplementary Figure S1 shows the accumulation of outcomes during 5-year follow-up. All outcomes were more common among long-term BZDRs users, and the difference developed mainly during the first 3 years.

The risk of all LMM outcomes was elevated for long-term use compared to short-term use (Table 2). Long-term use was associated with 27% (aHR 1.27, 95% CI 1.23–1.31) increased risk of LMM compared with short-term use. Concerning components of the LMM outcome, long-term use was associated with 42% (aHR 1.42, 95% CI 1.34–1.50) increased risk of disability pension, 26% (1.26, 1.20–1.32) increased risk of long-term unemployment, and 26% (1.26, 1.21.–1.31) increased risk of long-term sickness absence. The shortest mean time to event was observed for the combined outcome of LMM, 1,168 days for long-term users and 1,375 days for short-term users. These durations are 64.1 and 75.5% of the 5-years follow-up, respectively. The longest mean time to event was for DP, which on average, took place on fourth year of follow-up.

**Table 2.** Risk of labor market outcomes associated with long-term benzodiazepine and related drug use compared with short-term use during 5-years follow-up

	Mean time to event (SD)	No. of events	Unadjusted HR (95% CI)	Adjusted HR (95% CI)^a^
Risk of labor market marginalization (=DP, long–term unemployment or long-term sick leave)				
Short-term users	1,375 (650)	9,252	ref	ref
Long-term users	1,168 (716)	6,554	1.54 (1.50–1.59)	1.27 (1.23–1.31)
Risk of disability pension				
Short-term users	1,692 (388)	2,717	ref	ref
Long-term users	1,596 (498)	2,277	1.69 (1.60–1.78)	1.42 (1.34–1.50)
Risk of long–term unemployment (>180 days)				
Short-term users	1,498 (565)	4,646	ref	ref
Long-term users	1,308 (656)	3,496	1.62 (1.55–1.69)	1.26 (1.20–1.32)
Risk of long–term sick leave (>90 days)				
Short-term users	1,534 (568)	5,295	ref	ref
Long-term users	1,397 (659)	3,682	1.43 (1.37–1.49)	1.26 (1.21–1.31)

a Adjusted for age, gender, CV disease (hypertension, coronary artery disease, chronic heart failure, stroke), diabetes, rheumatoid arthritis, asthma/COPD, cancer, inflammatory bowel disease, epilepsy, schizophrenia, bipolar disorder, depression, anxiety disorder, substance use disorder, receipt of social benefits, previous sick leave, education, use of other medications 30 days before BZDR start: antidepressants, opioids, non-opioid analgesics, and muscle relaxants.

**Figure 1.**
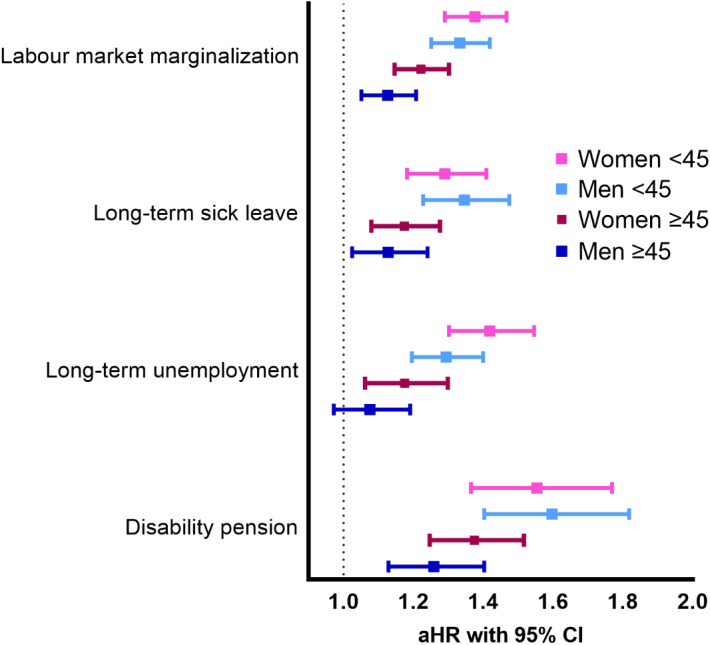
Risk of labor market outcomes associated with long-term benzodiazepine and related drug use compared to short-term use stratified by age (45 years) and sex.

When the study population was stratified according to age (45 years split) and sex, risk for all outcomes were somewhat higher for younger age groups in long-term use compared to short-term users (Figure 1).

**Figure 2.**
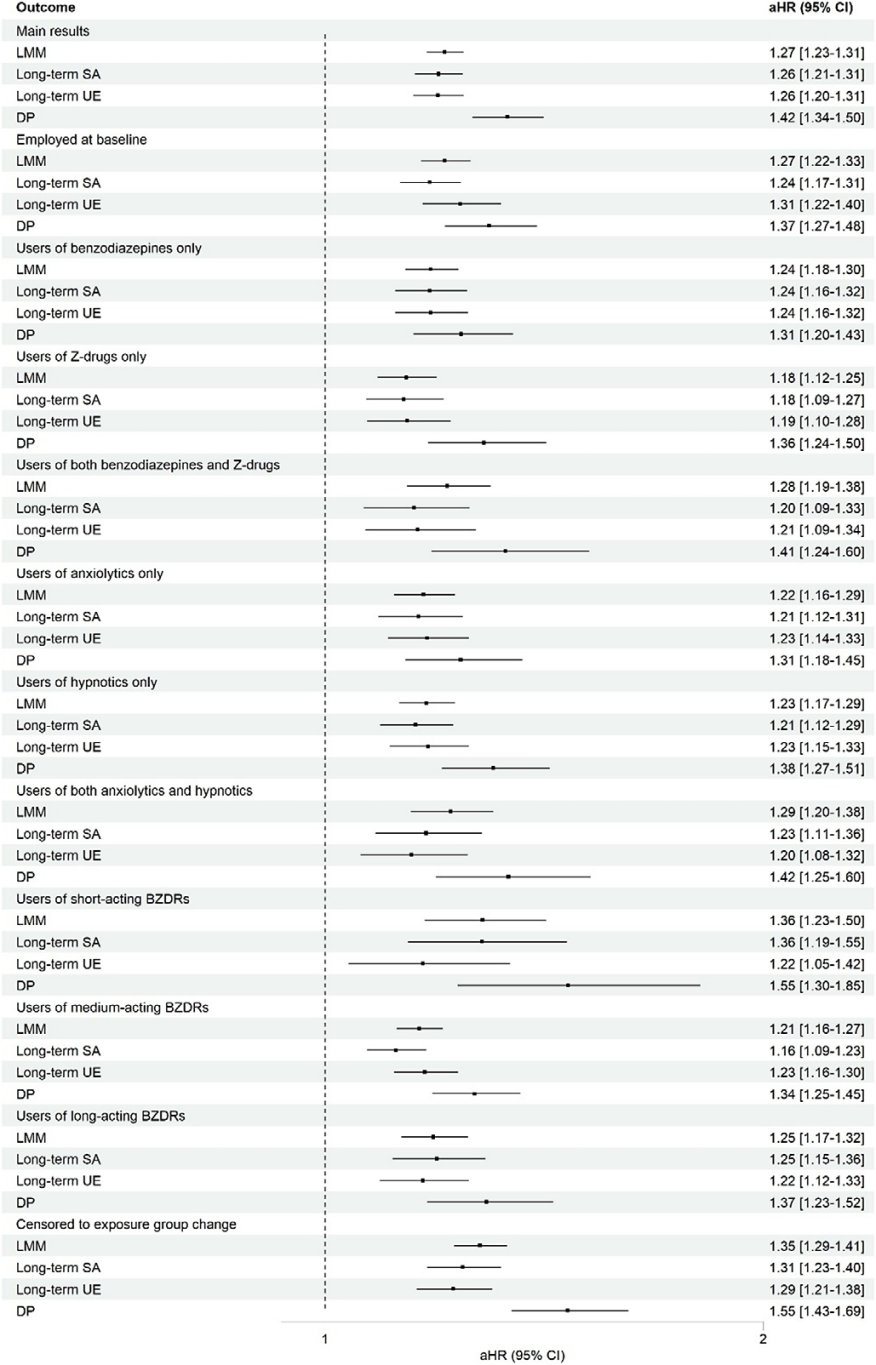
Sensitivity analyses on the risk of labor market marginalization associated with benzodiazepine and related drug (BZDR) use for those employed at baseline, stratified by initial drug class, type and half-life used, and by censoring to exposure group change. LMM, labor market marginalization; SA, sickness absence; UE, unemployment; DP, disability pension.

As sensitivity analysis, the population was stratified according to employment status at baseline, initial drug class, type and half-life, and lastly, by censoring at exposure group change (Figure 2). The results among those employed at baseline were similar to main analyses. The risk of LMM was rather similar between those who initiated with benzodiazepines only (aHR 1.24, 95% CI 1.18–1.30), Z-drugs only (1.19, 1.12–1.25) versus both (1.28, 1.19–1.38). The risks of LMM among anxiolytic-only users, hypnotic-only users and users of both anxiolytics and hypnotics were similar to each other and to the main analyses. aHRs stratified by half-life of BZDRs were similar to the main analysis for medium-acting BZDR users and long-acting BZDR users, whereas aHRs for LMM and DP were a bit higher among short-acting BZDR users, although confidence intervals were also wider. When censoring to exposure group changes, the risk of LMM was more pronounced (1.35, 1.29–1.41) than in the main analyses.

Disability pension was granted for N = 2,717 (11.0%) of the short-term users and for N = 2,277 (17.6%) of the long-term users (N = 4,994 disability pensions, 13.2% for the total cohort) (Supplementary Table S1). The most common diagnoses for disability pension were mental, behavioral, and neurodevelopmental disorders (ICD-10 category F), which represented 46% of DPs for short-term users and 55% of DPs for long-term users. Within that class, the most common second-level categories were F3 and specifically depression (Supplementary Table S2).

## Discussion

In this nationwide register-linkage study from Finland, new BZDR users were found to be at higher risk of LMM compared to short-term users. This was evident even after adjustments for sociodemographic background, somatic and psychiatric morbidity, receipt of social benefits, and use of other types of psychotropic medication. The increased risk was observed for all subcategories of LMM, namely disability pension, long-term sickness absence, and long-term unemployment. To the best of our knowledge, no previous studies have assessed the risk of BZDR use for LMM, also covering long-term sick leaves and long-term unemployment in addition to disability pensions. In addition, there is no previous research comparing the risk of LMM between long-term users and short-term users.

Our findings are in line with a previous Norwegian study, which reported a relatively high rate of granted disability pensions (11.2%) for new BZDRs users during 8 years of follow-up [15]. However, that study lacked a comparison group. In our study, 11.0% of short-term users were granted with DP whereas 17.6% of long-term users were granted with DP during 5 years of follow-up. The Norwegian study also included those who were dispensed only one package during 2006, and thus, the results are not fully comparable to our study, where we set a requirement of at least two dispensing of BZDRs.

We also showed that long-term users were at higher risk of all components of LMM, including long-term sickness absence and long-term unemployment, in addition to disability pension. The association between long-term BZDR use and risk of adverse labor market outcomes can potentially be linked to cognitive adverse effects of these drugs [10, 21, 22], or the underlying psychiatric conditions, or the combination of these both. Long-term treatment with BZDRs seems to be associated with problems in cognitive functioning, such as processing speed, sustained attention, memory function, problem-solving, and non-verbal and verbal memory. These are domains that most likely reduce the ability to seek and maintain a job. The causality between long-term BZDR use and cognition is not clear. However, a recent experimental study suggests that prolonged diazepam administration causes cognitive deficits via changes in synaptic dynamics in the brain. Such an effect could be due to diazepam-induced TSPO (translocation protein) activation and subsequent modulation of synapses by microglial cells [11]. These cognitive deficits overlap with those seen in anxiety or depressive disorders without BZDR use. It is possible that long-term BZDR use also has other effects, for example, impacting on motivation or the level of physical activity. In addition, chronic benzodiazepine use may reduce efficacy of antidepressant use, for example, through interference with neurogenesis [23], consequent lack of remission leading to LMM. However, there is solid evidence mainly on the cognitive effects of BZDRs [10, 21, 22], which can persist even after discontinuation of use [24].

The most common psychiatric disorders among long-term benzodiazepine users were substance use disorder, anxiety disorders, and affective disorders. In general, cognitive impairments caused by anxiety and affective disorders are milder than those caused by long-term benzodiazepine use [21, 25, 26]. The mean effect sizes (Cohen’s *d*) of cognitive deficits caused by long-term benzodiazepine use, major depression, and anxiety disorders range from 0.42 to 1.30, from 0.34 to 0.65, and from 0.02 to 0.44, respectively [27]. Cognitive deficits caused by long-term benzodiazepine use may be particularly detrimental for employment in persons whose cognitive reserve capacity is already impaired by their psychiatric disorder.

We found similar risks of LMM and DP for those who initiated the use of benzodiazepines, Z-drugs, or both. This is in contrast with a previous Norwegian study, which reported that Z-drug users had a lower risk and combination users had higher risk of DP than benzodiazepine users [15]. Similarly, we did not find major differences between anxiolytics versus hypnotics, or by elimination of half-life and risk of LMM. Our study also showed that the risk estimates of LMM outcomes were somewhat higher among younger age group (<45 years) compared to older age group, and thus, LMM can have larger relative effect on personal working life history among young adults than for those who are close to retirement. In contrast, the previous Norwegian study found higher age to be associated with DP among benzodiazepine users [15]. Although we adjusted for several important confounders, the possibility of residual confounding cannot be ruled out. It is possible that among younger persons, more severe conditions are concentrated on long-term users, whereas the groups may be more similar to each other within older age strata in terms of psychiatric comorbidity. However, making comparisons between long-term and short-term users reduced confounding by indication and by psychiatric morbidity in our study, as confounding would be much higher if the comparison group were generally healthy persons never using BZDRs.

In the light of these results, we need to consider why long-term BZDR use is so common (up to 34% in our study). The recent French study hinted that long-term use has been more acceptable and side effects less well known previously than today as older practitioners still prescribe more frequently long-term use [28]. Once treatment has been in use successfully for years, it is difficult to motivate either practitioner or patient to stop the treatment. The critical point seems to be initiation of BZDR use in the first place, and this should be the focus of any potential interventions aimed at preventing permanent decrease in job performance.

Psychiatric comorbidities, especially schizophrenia and substance use disorders, are associated with an increased risk of long-term BZDR use [7]. Both schizophrenia and substance use disorders are also more likely to lead to LMM. However, our analyses were adjusted for these conditions and BZDR use was still associated with the risk of LMM. We also excluded people who were already on disability pension, which represents the majority of persons with schizophrenia. [29] Frequently observed long-term BZDR use among patients with severe mental disorders is alarming and has been previously associated with impaired attention/working memory [22], and concomitant use of BZDRs with opioids, buprenorphine, or methadone can result into hazardous consequences such as an increased risk of mortality among concomitant users [30].

The generalizability of these results depends on the employment situation (e.g., unemployment rate), social security benefits and overall structures of labor market. The results are likely most generalizable to societies resembling that of Finland, namely other Nordic countries. The overall population employment rate was stable, without any major events such as economic recession and the employment rate was around 70% during the time frame used in this study. Thus, it is unlikely that drastic economical changes could have affected our results. Nevertheless, it is clear that working life is, on average, becoming more and more demanding for individuals with psychiatric disorders with or without BZDR use. It is possible that persons with mental disorders will need supported forms of employment to be able to attend working life. There is a trend that DPs are not granted in Finland as easily as previously, but such an effect would concern both long-term and short-term user groups in our study.

### Strength and limitations

We utilized large nationwide register-based data covering BZDR use and LMM outcomes, with relatively long follow-up time, stable employment rate, and overall economic situation in the country. For drug purchases, non-reimbursed purchases, mainly small packages of BZDRs, were not included in the register data and thus, the results may be underestimated, especially for short-term use. To further reduce uncertainty in exposure status, that is, whether BZDRs were actually used or not when prescribed, for example, for acute crisis, we included only those persons who had at least two consecutive purchases within first year. The follow-up time of 5 years is long enough to detect differences in LMM risks between initiated short-term versus long-term use of BZDRs drugs. The new user design was utilized to avoid prevalent user bias. Baseline information was equally collected for all cohort members. Sensitivity analyses were additionally censored to exposure group changes, namely change from short-term use to long-term use and vice versa and also to discontinuation of use. The results of these analyses showed somewhat similar results to the main analyses. We, however, lacked indications for BZDR prescriptions. The indication of use may be related to the duration of BZDR use and LMM outcomes. In addition, we did not have information on the severity of symptoms, and data on cognitive functioning was not available. The analyses were adjusted for important sociodemographic factors and comorbidities. However, register-based data does not cover all important factors related to, for example, substance use.

## Conclusions

Long-term use of BZDRs was associated with an increased risk of LMM. This affects especially younger people and thus, the individual and societal costs are large. The association may be partly explained by cognitive adverse effects of prolonged BZDR use. When prescribing BZDRs, cognitive adverse effects and their potential long-term consequences should be taken into account.

## Supporting information

Taipale et al. supplementary material Taipale et al. supplementary material

## Supplementary material

The supplementary material for this article can be found at http://doi.org/10.1192/j.eurpsy.2024.1745.
