# Supplementary material for: Long-term benzodiazepine use and risk of labor market marginalization in Finland: A cohort study with 5-year follow-up
Source: Eur Psychiatry. 2024 Apr 4;67(1):e34. doi: 10.1192/j.eurpsy.2024.1745 (PMC11059246; doi:10.1192/j.eurpsy.2024.1745)
Supplement: Taipale et al. supplementary material [file S0924933824017450sup001.docx]

Supplement to “Long-term benzodiazepine use and risk of labour market marginalization in Finland – A cohort study with five-year follow-up”, by Heidi Taipale, Antti Tanskanen, Terhi Kurko Tero Taiminen, Hanna Särkilä, Jari Tiihonen, Reijo Sund, Solja Niemelä, Leena Saastamoinen and Jarmo Hietala

| **Supplementary Table 1.** Distribution of diagnosis for granted disability pensions for short-term and long-term benzodiazepine and related drug users. | | | | | | | | | |
| --- | --- | --- | --- | --- | --- | --- | --- | --- | --- |
| **ICD-10** | **Description** | **Short-term users, N** | | **Long-term users, N** | | | | **chi-squared test p-value** | |
| A-B | Infectious and parasitic diseases | 7 | | <5 | |  | | | 0.322 |
| C-D | Neoplasms and anaemias etc. | 133 | | 77 | |  | | | 0.001 |
| E | Endocrine, nutritional and metabolic diseases | 28 | | 21 | |  | | | 0.700 |
| F | Mental, Behavioural and Neurodevelopmental disorders | 1244 | | 1246 | |  | | | <0.001 |
| G | Diseases of the nervous system | 186 | | 188 | |  | | | 0.070 |
| H | Diseases of the eye and adnexa | 28 | | 14 | |  | | | 0.111 |
| I | Diseases of the ear and mastoid process | 106 | | 78 | |  | | | 0.383 |
| J | Diseases of the respiratory system | 35 | | 27 | |  | | | 0.746 |
| K | Diseases of the digestive system | 37 | | 18 | |  | | | 0.055 |
| L | Diseases of the skin and subcutaneous tissue | 11 | | <5 | |  | | | 0.069 |
| M | Diseases of the musculoskeletal system and connective tissue | 738 | | 449 | |  | | | <0.001 |
| N | Diseases of the genitourinary system | 7 | | 7 | |  | | | 0.741 |
| Q | Congenital malformations, deformations and chromosomal abnormalities | 10 | | <5 | |  | | | 0.103 |
| R | Symptoms, signs and abnormal clinical and laboratory findings, not elsewhere classified | 9 | | 5 | |  | | | 0.458 |
| S | Injury, poisoning and certain other consequences of external causes | 97 | | 101 | |  | | | 0.126 |
| T | Injury, poisoning and certain other consequences of external causes | 35 | | 31 | |  | | | 0.823 |
| Z | Factors influencing health status and contact with health services | 5 | | 6 | |  | | | 0.551 |
| NA | Not specified/ recorded | <5 | | <5 | |  | | |  |
| Total |  | 2717 | | 2277 | |  | | |  |
| **Supplementary Table 2.** Distribution of diagnosis for granted disability pensions for short-term and long-term benzodiazepine and related drug users in ICD-10 subcategory F, referring to ‘Mental, Behavioural and Neurodevelopmental disorders’. | | | | | | | | | |
| **ICD-10 subcategories** | | | **Short-term users, N** | | **Long-term users, N** | | **chi-squared test p-value** | | |
| F0 Mental disorders due to known physiological conditions | | | 9 | | 16 | | | | 0.163 |
| F1 Mental and behavioural disorders due to psychoactive substance use | | | 34 | | 51 | | | | 0.066 |
| F2 Schizophrenia, schizotypal, delusional, and other non-mood psychotic disorders | | | 173 | | 189 | | | | 0.409 |
| F3 Mood [affective] disorders | | | 885 | | 840 | | | | 0.264 |
| F4 Anxiety, dissociative, stress-related, somatoform and other nonpsychotic mental disorders | | | 95 | | 99 | | | | 0.783 |
| F5 Behavioural syndromes associated with physiological disturbances and physical factors | | | <5 | | <5 | | | | 0.707 |
| F6 Disorders of adult personality and behaviour | | | 22 | | 28 | | | | 0.399 |
| F7 Intellectual disabilities | | | 10 | | 7 | | | | 0.465 |
| F8 Behavioural and emotional disorders with onset usually occurring in childhood and adolescence | | | 11 | | 5 | | | | 0.133 |
| F9 Unspecified mental disorder | | | <5 | | 7 | | | | 0.096 |
| Total | | | 1244 | | 1246 | | | |  |

**Supplementary Figure 1.** Cumulative incidence function curve for labour marker marginalization (A), disability pension (B), long-term sickness absence (C) and long-term unemployment (D) for long-term vs. short-term benzodiazepine and related drug users.

| 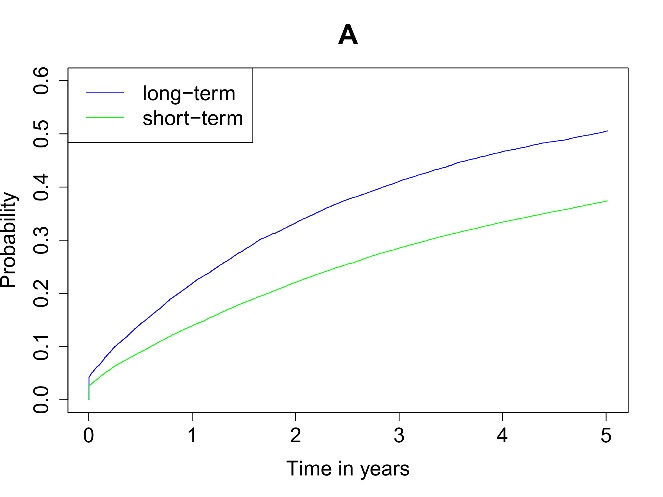 | 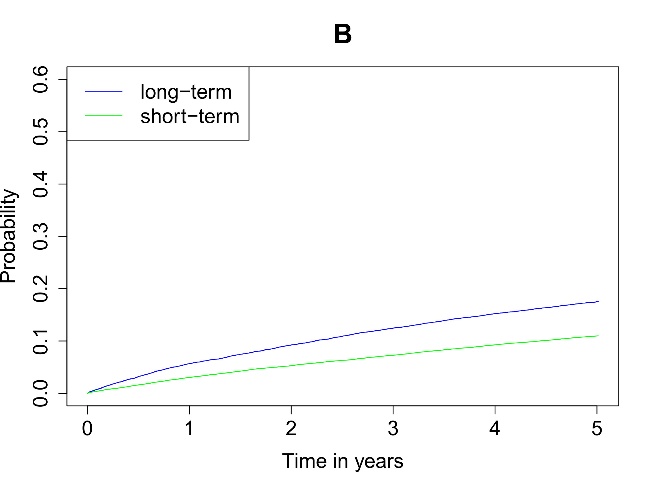 |
| --- | --- |
| 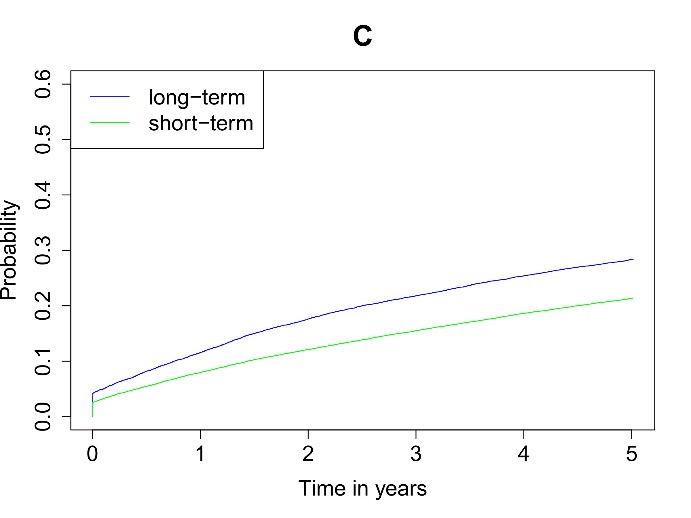 | 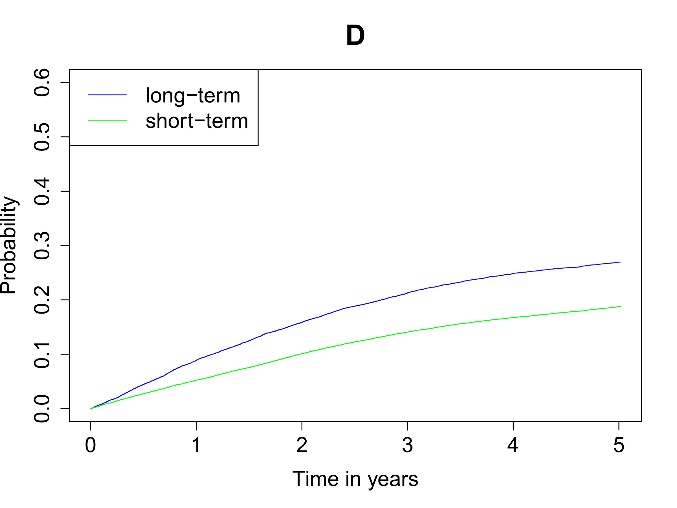 |
